# Seasonal and Spatial Distribution Characteristics of *Sepia esculenta* in the East China Sea Region: Transfer of the Central Distribution from 29° N to 28° N

**DOI:** 10.3390/ani14101412

**Published:** 2024-05-08

**Authors:** Min Xu, Linlin Yang, Zunlei Liu, Yi Zhang, Hui Zhang

**Affiliations:** 1 Key Laboratory of East China Sea Fishery Resources Exploitation, Ministry of Agriculture and Rural Affairs, Shanghai 200090, China; xumin@ecsf.ac.cn (M.X.); liuzl@ecsf.ac.cn (Z.L.); zhangyi@ecsf.ac.cn (Y.Z.); zhangh@ecsf.ac.cn (H.Z.); 2 East China Sea Fisheries Research Institute, Chinese Academy of Fishery Sciences, Shanghai 200090, China

**Keywords:** *Sepia esculenta*, seasonal variation, spatiotemporal distribution, East China Sea region, fishery management, average individual weight, global warming, golden cuttlefish

## Abstract

**Simple Summary:**

Since the 1990s, the golden cuttlefish (*Sepia esculenta*) has become the dominant cuttlefish fisheries target in the seas around China. In this paper, we aim to determine the current resource status and spatio-seasonal variation in the population in the East China Sea region. We found more juveniles at latitudes of 27.50–28.00° N and 29.00° N and more subadult individuals at 28.50° N in autumn, exhibiting different growth rates and resource densities. In addition, we found the majority of the catches were composed of parent groups in spring, while in autumn, the majority of the catches were composed of juvenile groups. We concluded that the subadult groups might have dispersed widely for feeding and growth along the latitude of 30.00° N and to the south in summer, and the southern area of the Yangtze River extending north was the spawning ground in spring. The groups of *S. esculenta* preferred to stay in areas with a stable water temperature of ~20.00 °C, and many *S. esculenta* juveniles might have adapted to endure the negative influence of the low oxygen content in summer. The depth range of the *S. esculenta* population was 10.00–133.00 m from spring to autumn, but this shrank to 66.00–107.00 m in winter.

**Abstract:**

The golden cuttlefish (*Sepia esculenta*) is an important cephalopod species with a lifespan of approximately one year. The species plays a crucial role in marine ecological support services and is commercially valuable in fisheries. In the seas around China, this species has emerged as the main target for cuttlefish fisheries, replacing *Sepiella maindroni* since the 1990s. Variations in oceanographic conditions associated with global warming could significantly impact the temporal-spatial distribution of the species. In this study, we performed bottom trawling surveys with four cruises during 2018–2019 in the East China Sea region to determine the current resource status and seasonal-spatial variations in *S. esculenta*. We found that the average individual weight (AIW) values were 4.87 and 519.00 g/ind at stations located at 30.50° N, 124.00° E and 30.50° N, 124.50° E, respectively, with the aggregation of larvae and parent groups in spring. The species was not distributed north of 32.00° N in summer. The catch per unit effort by weight (CPUE_w_) value decreased in the order of 2772.50→2575.20→503.29→124.36 g/h, corresponding to latitudes of 34.50° N→34.00° N→33.50° N→32.50° N 121.50° E in autumn. The most suitable fishing areas were the south of the East China Sea region in spring; the south of the East China Sea region extending to the center and outer parts of the East China Sea region in summer; the south of the Yellow Sea close to the Haizhou Bay fishing ground and the forbidden fishing line region of the Lusi and Dasha fishing grounds in autumn; and the south and center of the East China Sea region in winter. The most suitable sea bottom temperature (SBT) values from spring to winter were 14.76–20.53 °C, 19.54–22.98 °C, 11.79–17.64 °C, and 16.94–20.36 °C, respectively. The most suitable sea bottom salinity (SBS) values were 31.53–34.80‰ in spring, 32.95–34.68‰ in summer, 31.51–34.77‰ in autumn, and 33.82–34.51‰ in winter. We concluded the following: (1) the southern and northern areas of the East China Sea region are spawning and nursery grounds, respectively, in spring; (2) the central distribution is located at a latitude of 28.00° N in autumn and winter; and (3) the southern area of the Yangtze River to the north is a spawning ground in spring, and the areas located at 29.00–34.50° N, 124.00–124.50° E, and 28.00–30.50° N, 125.50–126.50° E are nursery grounds. The results of this study provide useful guidance for appropriate fisheries management, thereby avoiding a collapse in the *S. esculenta* population, which has been experienced in other species in this area.

## 1. Introduction

Cephalopods, including squids, cuttlefish, and octopus, are important providers of marine ecological support services and commodities in commercial fisheries [1]. They are the main prey items for many seabirds, larger fishes, and marine mammals [2]. Their marine catch has been increasing globally at unprecedented rates in recent decades [3], although some of their stocks have been overexploited [4]. In China, coastal cephalopods are a vital component of the local fisheries catch [5], with a noticeable and substantial increase in the total biomass in recent decades [6]. Their production fluctuations are likely to be largely driven by environmental variations [7]. Variations in oceanographic conditions associated with global warming variations could significantly influence the temporal-spatial distribution of cephalopods [8]. In the seas around China, the golden cuttlefish (*Sepia esculenta*) has become the dominant cuttlefish fisheries target, replacing *Sepiella maindroni* since the 1990s [9]. Yan et al. (2007) reported that the population of *S. esculenta* was highest in autumn and lowest in summer, and it was the dominant cephalopod species in the East China Sea from 2008 to 2009 [10].

*Sepia esculenta*, commonly known as ‘moyu’ in Chinese slang, belongs to the phylum Mollusca (Cephalopoda, Sepioidea, Sepiidae, Sepia), and is the most important commercial coastal cepholopod species in Japan, Korea, and China [10]. The species inhabits warm-temperate shallow coastal sea at depths of ~10–100 m, e.g., the Russian far east region, the waters west and south of the Korean Peninsula, coastal sea areas of south Japan, the seas around China, and the Philippine islands in the Northwestern Pacific [11]. Their traditional main fishing grounds include Hinoshino Beach and Seto Inland Sea of Japan, Lanshantou, Qingdao, the central and northern part of the Yellow Sea in China, and Jeju Island in Korea [12]. The species has a short lifespan of one year or less and conducts batch spawning (egg maturation in batches) with a trophic level of 2.86 [13]. The adult carcass length can be ~20 cm [14]. They are a nektobenthic migratory species and are the main target of cage fishing and a bycatch target of trawling and fixed net fishing [11]. The female groups prefer to lay individual egg capsules, each laying one egg capsule on the branches of macroalgae or the sea floor and producing 23–65 egg capsules during a single egg-laying period [15]. Newly hatched juveniles are miniature adults that already have schooling and nektobenthic tendencies [16] and usually prefer to remain on the sea bottom [17]. Their spawning sites and distribution are usually influenced by prey availability and macroalgae attachments during the migration [18]. Li (1963) argued that grouping time has a close relationship with the population number along the Zhejiang coast [19]. As a carnivorous species, they prey on a variety of fish and crustacean species, such as the fish *Engraulis japonius*, *Setipinna taty*, and *Collichthys lucidus*, and crustaceans of the Xanthidae, including *Squilla oratoria*, *Trachypenaeus curvirostris*, and *Acetes*, as well as other *S. esculenta* individuals [20,21,22]. Dong et al. (1991) [12] and Zheng et al. (2003) [23] identified 50.0% crustaceans, 25.0% fish, and 25.0% *S. esculenta* in the diet of *S. esculenta* in the Yellow Sea. Natsukari and Tashiro (1991) concluded that the diet consisted of 45.7% fish, 39.5% crustaceans in Order Decapoda, 8.0% Stomatopoda and Cephalopoda, and 0.7% Mysidacea [24].

Robinson et al. (2013) found that the population of *S. esculenta* tended to be distributed in cold areas and preferred a relatively low optimal water temperature in the Japan Sea and Korean waters [25]. Warmer water might negatively affect the growth and development of the species [25]. Negative correlations between catches and the increasing sea surface temperature (SST) were observed in autumn and winter [26]. Belkin (2009) observed an increase of 1.22 °C in the SST in the East China Sea from1982 to 2006, which was potentially associated with global warming [27,28,29]. The winter SST in the East China Sea has tended to increase since the late 1980s, which might significantly accelerate the somatic growth rate and cause advanced maturation at small sizes and younger ages [30,31,32]. Pang et al. (2018) explored the impacts of environmental changes on Chinese coastal cephalopods in the seas around China and concluded that fluctuations in coastal productions were mainly driven by large-scale environmental variations associated with climate change and/or marine ecosystem regime shifts [26]. The *S. esculenta* migrated to the overwintering ground in China’s Yellow Sea, where the water temperature was gradually decreasing [26]. Crozier and Hutchings et al. (2014) found that when there was a higher water temperature in the overwintering area, the overwintering period was shortened [33]. This induced the early onset of spawning migration, resulting in a relatively poor reproductive condition, high marine mortality, and subsequently weak recruitment [33]. It is, therefore, necessary to understand the resource status and spatio-temporal variation characteristics of *S. esculenta* in recent years.

The aim of this study was to determine the current resource status and spatio-seasonal variations in the *S. esculenta* population with a detailed investigation in the East China Sea region. We also compared our data with historical distribution records to obtain a clear picture of the movement of the resource center under the warming of the East China Sea region. We also attempted to identify the location of the spawning and nursery grounds, the most suitable range area, and the impact of environmental factors on the population. The results will enable the impacts of environmental variations on population fluctuations to be determined. They will also provide useful guidance for appropriate fisheries management, thereby avoiding a collapse in the *S. esculenta* population, which has been experienced in other species in this area.

## 2. Materials and Methods

We conducted four consecutive bottom trawling surveys with the survey vessels (#Zhongkeyu 211 and 212) in cruises during 2018–2019 in the East China Sea region, which covered the southern Yellow Sea and the East China Sea (autumn: 2–11 November 2018; winter: 4–27 January 2019; spring: 22 April to 10 May 2019; summer: 13 August to 27 September 2019) (Table 1). The trawling net had a mouth size of 102 mesh with a height of 10–15 m. The headline was 72.24 m long, and the bottom line was 82.44 m long. The net had a mesh size of 20.00 mm. Within the survey area (26°30′–35°00′ N, 120°00′–127°00′ E), survey stations were determined using a sampling grid with dimensions of 30 min of latitude and 30 min of longitude (30′ × 30′). The in situ survey in all seasons is performed by adopting a snake-like pattern along the route. In the southern area below 30°00′ N latitude, the survey was performed from north to south. In the northern area above 30°00′ N latitude, the survey was conducted from south to north. Following the surveys, fish, crustaceans, and other organisms, including *S. esculenta*, were identified to the lowest possible taxonomic level, counted, and weighed to the nearest 0.1 g of wet weight in the laboratory.

The biomass index representing catch density per unit of time is measured using two components: biomass density (unit: g·h^−1^) and individual density (unit: ind·h^−1^). An SBE-19 conductivity-temperature-depth (CTD) instrument (SeaBird-Scientific, Bellevue, WA, USA) was used at each site to record hydrographic parameters, such as depth, water temperature, salinity, and the dissolved oxygen (DO) concentration. Among the parameters measured, SST, sea surface salinity (SSS), and sea surface dissolved oxygen (SSDO) were selected to define the mean temperature, salinity, and DO of the water layer within 3 m of the surface, and sea bottom temperature (SBT), sea bottom salinity (SBS), and sea bottom dissolved oxygen (SBDO) were selected to define the mean temperature, salinity, and DO of the water layer within 2 m of the bottom for depths < 50 m, or the water layer within 2−4 m of the bottom for depths > 50 m.

The formula for calculating catch per unit effort (CPUE) was as follows:

$${CPUE}_{n}=\frac{N_{i}}{t_{i}}$$

$${CPUE}_{w}=\frac{W_{i}}{t_{i}}$$

where $N_{i}$ is the catch in number (ind) at *i* station; $W_{i}$ is the catch in weight (g) at *i* station; and $t_{i}$ is the trawling time (h) at *i* station. Additionally, we defined the average individual weight (AIW) as the ratio of the CPUE by weight (CPUE_w_) against CPUE by number (CPUE_n_) at a station.

The suitability index (*SI*) was used to assess the habitat suitability of *S. esculenta* according to the functional relationships between environmental factors and resource abundance. The biomass data of this species varied across the different seasons due to the variations in the proportions of the population in the different stages of their life histories. There can be large differences in size among these individuals, and therefore, we used the number of individuals caught per unit to construct the *SI*. The relationships between environmental variables (depth, SST, SBT, SSS, SBS, SSDO, and SBDO) and *SI* were fitted using a smooth function with a range of 0.0–1.0. An *SI* value closer to 1.0 means a higher suitability index, and an *SI* value closer to 0.0 means a lower suitability index. *SI* values between 0.7 and 1.0 corresponded to environmental factors that were considered the most suitable environmental range. An *SI* value of 0.7–1.0 was most suitable, with relative suitability at values of 0.4–0.7 and instability at values of 0.0–0.4 [34].

The *SI* was calculated as shown below:

$$SI=\frac{\hat{Y}-{\hat{Y}}_{min}}{{\hat{Y}}_{max}-{\hat{Y}}_{min}}$$

where $\hat{Y}$ is the catch in number per unit area after a smoothed regression, and ${\hat{Y}}_{max}$ and ${\hat{Y}}_{min}$ are the maximum and minimum predicted values, respectively.

The *HSI* model was composed of the *SI* index for all the environmental variables. The model used a boosted regression tree (*BRT*) and the total variance contribution rate approach to calculate the weight of environmental factors. We calculated *HSI* values by an arithmetic mean model (*AMM*) using the equation below [35]:

$${HSI}_{AMM}=\frac{1}{\sum_{i=1}^{n}\omega_{i}}\cdot \sum_{i=1}^{n}{SI}_{i}\omega_{i}$$

where *HSI* is the habitat suitability index, *SI_i_* is the *SI* value of the environmental variable *I*, $\omega_{i}$ is the weight of the environmental variable *i*, and *n* is the number of environmental factors.

## 3. Results

### 3.1. Seasonal Characteristics of the Distribution, CPUE_w,_ and AIW

**Figure 1:**
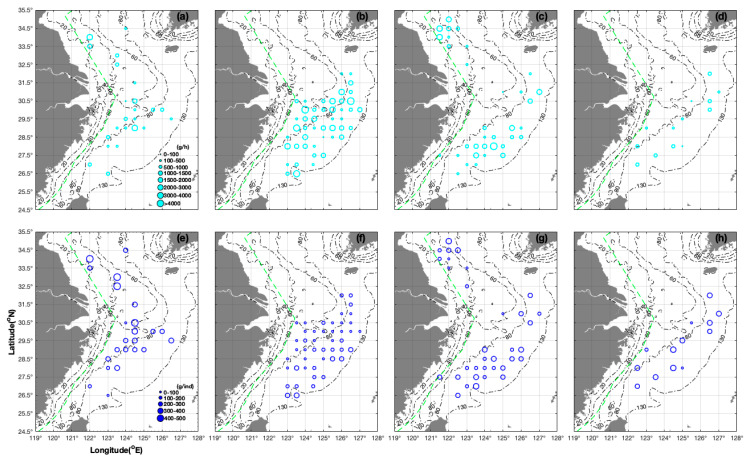
Seasonal distribution characteristics of CPUE_w_ (unit: g/h) classified by cyan group (0–100, 100–500, 500–1000, 1000–1500, 1500–2000, 2000–3000, 3000–4000, and >4000 g/h) and AIW (unit: g/ind) classified by blue group (0–100, 100–200, 200–300, 300–400, and 400–500 g/ind). The size of the values is represented by hollow circles. The depth gradient (20–130 m) is represented by a black chain line. (**a**) CPUE_w_ in spring; (**b**) CPUE_w_ in summer; (**c**) CPUE_w_ in autumn; (**d**) CPUE_w_ in winter; (**e**) AIW in spring; (**f**) AIW in summer; (**g**) AIW in autumn; and (**h**) AIW in winter.

The whole survey area could be divided into two regions in spring: region I 32.50–34.50° N, 122.00–124.00° E, with an SBT of 9.60–15.64 °C and SBS of 30.56–33.72‰; and region II 26.50–31.50° N, 122.00–126.50° E, with an SBT of 11.59–22.79 °C and SBS of 28.95–35.25‰. The station at 34.50° N, 124.00° E, which was the northernmost survey station and was located adjacent to the Korean Peninsula, had a CPUE_w_ of 250.62 g/h and AIW of 200.50 g/ind under conditions with an SBT of 9.60 °C and SBS of 32.76‰ in spring (Figure 1a,e). In region I, the regional average CPUE_w_ and AIW were 1125.08 g/h and 500.24 g/ind, respectively. In region II, the regional average CPUE_w_ and AIW were 561.47 g/h and 262.14 g/ind, respectively, i.e., less than half that of region I. The average CPUE_w_ (1931.90 g/h) at the stations at 34.00° N, 122.00° E and 33.50° N, 122.00° E was 1176.40 g/h higher than the average value (735.02 g/h) at the stations at 33.00° N, 123.50° E and 32.50° N 123.50° E (Figure 1a), but the average AIW (415.35 g/ind) was lower at the longitude of 122.00° E at 33.50–34.00° N than the average value (735.02 g/ind) at 123.50° E in 32.50–33.00° N (Figure 1e). The AIW values were 4.87 and 519.00 g/ind in the stations at 30.50° N, 124.00° E and 30.50° N, 124.50° E, respectively, which was due to the group aggregation of larvae at an SBT of 16.81 °C and SBS of 33.00‰, and parent groups at an SBT of 13.67 °C and SBS of 33.41‰ (Figure 1e). The CPUE_w_ and AIW ranged from 802.80–812.90 g/h and 89.20–109.85 g/ind over the spatial range of 26.50–27.00° N, 122.00–123.00° E, with an SBT of 18.60−20.53 °C and SBS of 34.40–34.52‰. The CPUE_w_ ranged from 201.94 to 2184.70 g/h, and AIW ranged from 188.85to 414.89 g/ind in the range of 28.00–30.00° N, 123.00–126.50° E with an SBT of 14.27–19.93 °C and SBS of 33.61–34.77‰. The AIW value varied from 414.89 to 273.09 g/ind over the spatial range of 30.00° N→29.00° N, 124.50° E (Figure 1e), and the CPUE_w_ value was 2184.70 g/h at 29.00° N, 124.50° E with an SBT of 18.13 °C and SBS of 34.77‰ (Figure 1a).

The whole survey area could also be divided into two regions in summer: region I, 29.50–32.00° N with an SBT of 13.09–26.97 °C and SBS of 31.30–34.65‰; and region II south of 29.50° N with an SBT of 17.23–28.50 °C and SBS of 32.31–34.68‰. We found no distribution of *S. esculenta* north of 32.00° N, including the regions north of 31.00–32.00° N, west of 126.00° E; 29.00–30.50° N, west of 123.50° E; and 28.00–29.00° N, west of 123.00° E (Figure 1b,f). The regional average CPUE_w_ and AIW in the whole area were 1297.94 g/h and 117.08 g/ind, respectively. The AIW value decreased from 140.00 to 77.00 g/h at the station at 32.00° N→31.00° N 126.00° E (Figure 1f). The CPUE_w_ and AIW varied from 615.17 to 6678.00 g/h and 90.60 to 39.05 g/ind over the spatial range of 30.50° N 123.50° E→126.50° E, indicating that more juveniles were present in the offshore area (Figure 1b,f). The AIW value was 177.00 g/ind in the station at 30.50° N, 125.00° E (Figure 1f). The CPUE_w_ and AIW decreased from 5200.20 to 1180.40 g/h and 96.30 to 75.66 g/ind at the latitude of 30.00° N and longitudes of 124.00° E to 127.00° E. The AIW was in the range of 68.14 to 118.10 g/ind at the latitude of 29.50° N, and the CPUE_w_ was highest (1561.09 g/h) at 29.50° N, 124.50° E with an SBT of 21.25 °C and SBS of 34.37‰ (Figure 1b,f). At the latitude of 29.50° N, the AIW at 124.00° E was twice that at 124.50° E (112.67 g/ind vs. 68.14 g/ind), and the CPUE_w_ was 1062.92–2510.13 g/h at 29.00° N, 125.00–125.50° E. At 29.00° N, 123.50° E→126.50° E, the CPUE_w_ and AIW decreased from 3794.70 to 687.27 g/h and increased from 58.38 to 210.17 g/ind. At 28.50° N, 124.00° E→125.50° E, the CPUE_w_ decreased from 1780.52 to 413.17 g/h, and the AIW increased from 63.59 to 223.33 g/ind. At 28.00° N, 123.00° E→124.50° E, the CPUE_w_ decreased from 2910.47 to 361.20 g/h, and the AIW increased from 61.82 to 120.40 g/ind (Figure 1b,f).

The whole survey area could also be divided Into two regions in autumn: region I 32.50–35.00° N, 121.50–123.00° E, with an SBT of 9.52–19.45 °C and SBS of 30.84–33.23‰; and region II 26.50–32.00° N, 121.50–127.00° E with an SBT of 17.35–23.15 °C and SBS of 32.31–35.07‰. The regional average CPUE_w_ and AIW values of regions I and II were 1118.63 g/h and 179.88 g/ind and 852.23 g/h and 225.45 g/ind, respectively. The CPUE_w_ decreased in the order of 2772.50→2575.20→503.29→124.36 g/h, which corresponded to the latitudes of 34.50° N→34.00° N→33.50° N→32.50° N, 121.50° E (Figure 1c). In region II, the CPUE_w_ increased from 88.24 to 1996.08 g/h at 31.00° N, 125.00° E→127.00° E, with an average value of 783.32 g/h (Figure 1c), and the AIW value increased from 74.78 to 243.71 g/ind at 31.00° N 125.00° E→126.00° E (Figure 1g). For all stations at the latitudes of 29.00° N and 28.50° N, the highest CPUE_w_ values were 1696.49 g/h at 29.00° N, 125.50° E and 934.50 g/h at 28.50° N, 124.50° E, which corresponded to average values of 848.50 and 606.45 g/h at the latitudes of 29.00° N and 28.50° N, respectively (Figure 1c). The CPUE_w_ increased from 796.40 to 4346.00 g/h at 28.00° N, 123.00° E→124.50° E, with an average value of 1879.10 g/h (Figure 1c). The CPUE_w_ value increased from 208.00 to 1246.00 g/h at 27.50° N, 121.50° E→125.00° E with an average value of 681.04 g/h. The average CPUE_w_ value was 340.15 g/h at the stations at 27.00° N, 123.00° E and 27.00° N, 123.50° E (Figure 1c).

**Figure 2:**
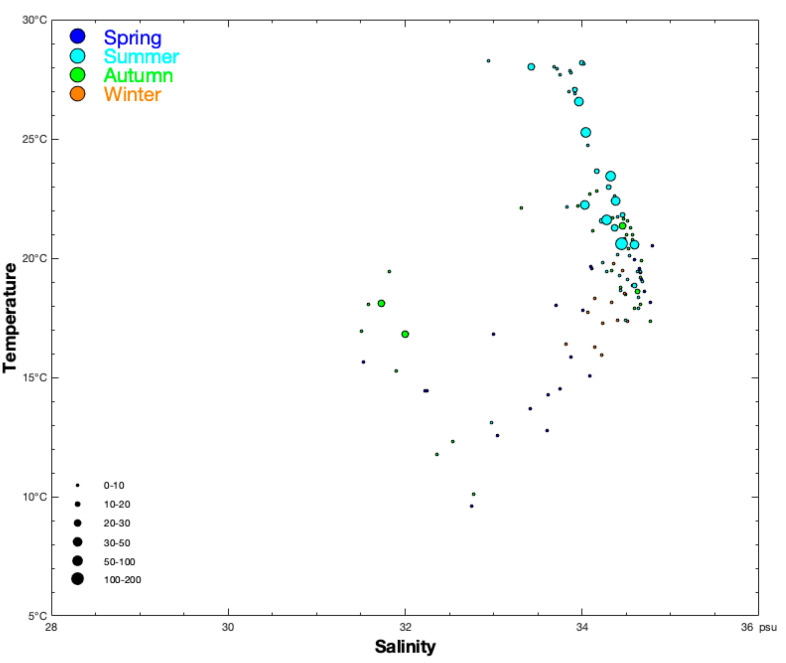
Relationship between sea bottom salinity (SBS, unit: psu) and sea bottom temperature (SBT, unit: °C) for CPUE_n_ sizes classified by group (0–10, 10–20, 20–30, 30–50, 50–100, and 100–200 ind/h). The data in spring, summer, autumn, and winter are denoted by blue, light sky blue, green, and brown-red solid circles, respectively.

The regional average CPUE_w_ and AIW values were 504.45 g/h and 291.96 g/ind, respectively, at 27.00–32.00° N, 122.50–127.00° E, with an SBT of 11.86–21.55 °C and SBS of 32.57–34.66‰ in winter (Figure 1d,h). The regional average CPUE_w_ of the northern survey area at 29.50–32.00° N, 125.00–127.00° E, with an SBT of 13.50–19.20 °C and SBS of 33.07–34.40‰, was 555.97 g/h, which was slightly higher than the value of 460.29 g/h in the southern area at 27.00–29.00° N, 122.50–125.00° E, with an SBT of 15.93–21.55 °C and SBS of 34.14–34.66‰ (Figure 1d). There was a seasonal order in the average CPUE_w_, the upper limit of CPUE_w_, and average CPUE_n_ for all survey stations—summer (1297.94 g/h, 6678.00 g/h, 17.40 ind/h) > autumn (922.75 g/h, 4346.00 g/h, 5.63 ind/h) > spring (683.99 g/h, 2705.00 g/h, 2.93 ind/h) > winter (504.45 g/h, 932.36 g/h, 1.64 ind/h), which differed from the order of AIW—spring (313.91 g/ind) > winter (291.96 g/ind) > autumn (213.39 g/ind) > summer (117.08 g/ind) (Figure 1 and Figure 2, Table 2).

### 3.2. The Population Numbers and Areas Most Suitable for Fishing

Group sizes of 0–100 g/ind were most commonly observed (24 stations) in summer and were least frequent (2–4 stations) in the other three seasons. Group sizes of 100–200 g/ind were only observed at two stations in spring and winter and 12–15 stations in summer and autumn. The frequency of observed group sizes of 200–300 g/ind followed the order of autumn and spring (10–11 stations) > summer (6 stations) > winter (2 stations). Group sizes of 300–400 and 400–500 g/ind were observed at one and zero stations, respectively, in summer, and 4–6 and 1–2 stations, respectively, in the other three seasons. Group sizes of >500 g/ind were only found at four stations in spring (see Table 2).

**Figure 3:**
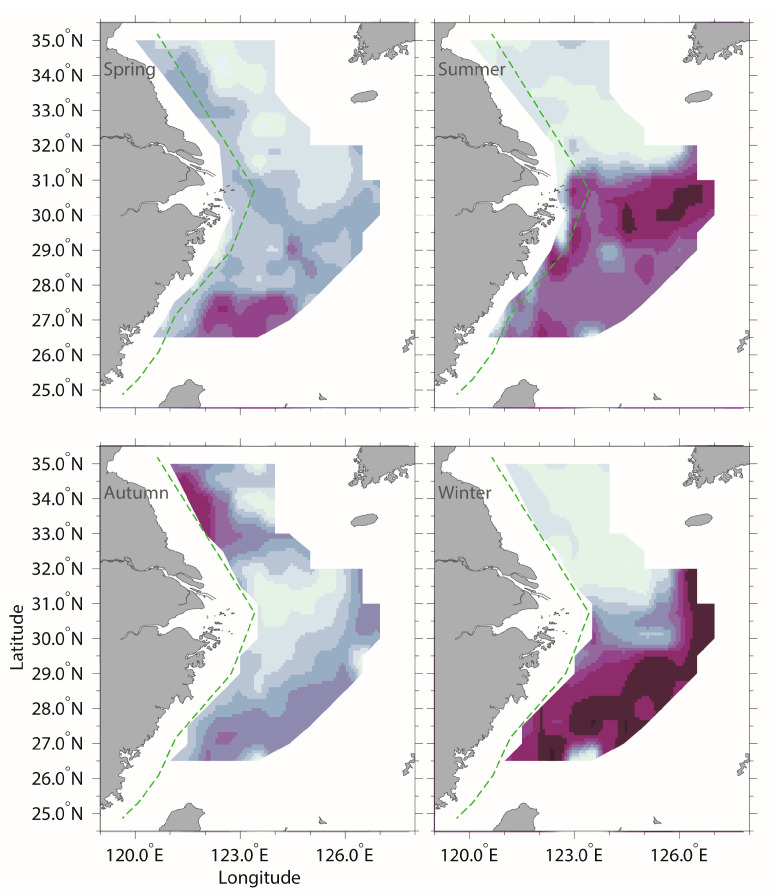
The bottom horizontal distributions of the habitat suitability index (HSI) of the golden cuttlefish (*Sepia esculenta*) during the various surveys. The black dots indicate sampling stations.

The most suitable areas for fishing in spring were the south of the East China Sea region (26.50–27.50° N, 122.00–124.00° E), including the areas of 27.50° N, 122.50–124.00° E; 27.00° N 122.00–123.50° E; and 26.50° N, 123.00–123.50° E (Table 2 and Figure 3). The most suitable areas for fishing in summer were the south of the East China Sea extending to the central area, and outer part of the East China Sea (26.50–31.00° N, 122.00–127.00° E), including the areas of 30.50−31.00° N, 123.00−127.00° E; 30.00° N, 124.50–127.00° E; 29.50° N, 123.00–126.50° E; 29.00° N, 122.33–123.00° E; 28.50° N, 122.50–125.00° E; and 26.50° N, 122.00–122.50° E (Table 2 and Figure 3). The most suitable areas for fishing in autumn were the south of the Yellow Sea close to the Haizhou Bay fishing ground and the forbidden fishing line parts of the Lusi and Dasha fishing grounds (33.00–34.50° N 121.50−122.00° E) (Table 2 and Figure 3). The most suitable areas for fishing in winter were the south and center of the East China Sea (26.50–32.00° N, 121.00–127.00° E), including the areas of 31.50–32.00° N, 126.50° E; 31.00° N, 126.00–127.00° E; 30.50° N, 126.50–127.00° E; 30.00° N, 126.50° E; 29.50° N, 124.00–126.50° E; 29.00° N, 123.50–126.00° E; 28.50° N, 124.00–126.00° E; 28.00° N, 122.50–125.00° E; 27.50° N, 122.00–125.00° E; 27.00° N, 121.50–124.00° E; and 26.50° N, 121.00° N–121.50° E (Table 2 and Figure 3).

### 3.3. The Environmental Factors Supporting S. esculenta Populations

**Figure 4:**
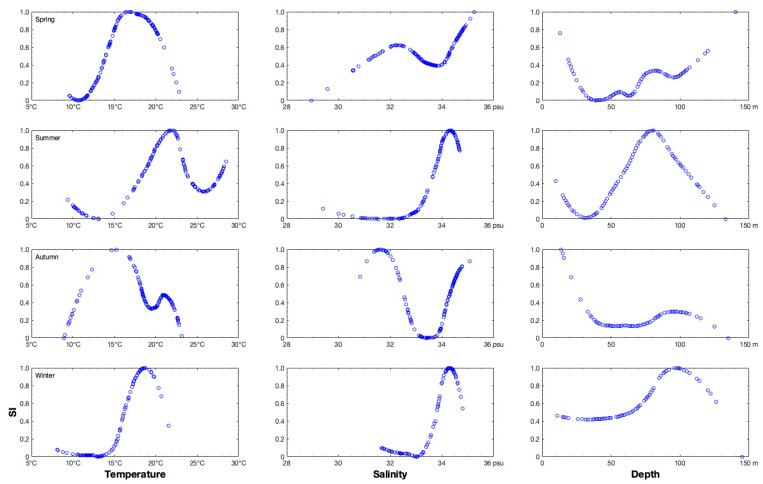
The variations in suitability index (SI) values (0.0–1.0) denoted by blue hollow circles for bottom temperature (range: 5–30 °C), bottom salinity (range: 28–36 psu), and depth (range: 0–150 m) in spring, summer, autumn, and winter.

The depth range of the *S. esculenta* population was 10.00–133.00 m from spring to autumn, but this shrank to 66.00–107.00 m in winter (Table 3). The most suitable depth with an SI > 0.7 was 140.00 m in spring, 65.00–96.00 m in summer, 14.00–21.00 m in autumn, and 77.00–122.00 m in winter (Figure 4). The range of SSTs (13.17–25.78 °C in spring; 16.91–25.16 °C in autumn) and SBTs (9.60–20.53 °C in spring; 10.08–22.83 °C in autumn) were similar in spring and autumn, respectively, and SSTs were 3.00–6.00 °C higher than SBTs (Table 3). The range of SBTs was larger than that of SSTs in summer (SST 26.20–29.61 °C; SBT 13.09–28.25 °C), and the upper limits of SST and SBT were close in summer (29.61 °C vs. 28.25 °C) (Table 3). The lower limit of SBT in winter was higher than in spring and autumn (15.93 vs. 9.60–10.08 °C), and the upper limit of SBT (19.76 °C) was close to the values in spring (20.53 °C) and autumn (22.83 °C) (Table 3). The most suitable SBT values (SI > 0.7) in spring, summer, autumn, and winter were 14.76–20.53 °C, 19.54–22.98 °C, 11.79–17.64 °C, and 16.94–20.36 °C, respectively (Figure 4). The second most suitable SBT ranges for fishing (SI = 0.4−0.7) were 25.99–28.50 °C in summer and 19.33–22.20 °C in autumn (Figure 4). The ranges of SSS and SBS were 31.00–35.00‰ in spring and autumn, which demonstrated the influence of dilution with water from the Changjiang River on bottom salinity. The SBS range was 32.95–34.68‰, revealing an influence from the East China Sea warm current in summer and winter (Table 3). The SSS range of 27.69–34.23‰ in summer was clearly higher than in spring and autumn, and the upper limit of SSS (34.23‰) in summer was close to that of spring (34.52‰) and autumn (34.45‰). The overwintering group preferred a high SSS value of 33.68–34.39‰ in winter (Table 3). The most suitable SBS values were 31.53–34.80‰ in spring, 32.95–34.68‰ in summer, 31.51–34.77‰ in autumn, and 33.82–34.51‰ in winter (Figure 4). The SBT and SBS ranges were 18.87–28.19 °C and 33.43–34.59‰ in summer when the CPUE_n_ was 11.00–171.00 ind/h (Figure 2), which represented a wide range of water temperatures and a narrow range of salinities. In the group where the CPUE_n_ was 15.85–29.00 ind/h, the SBS range was wider (31.73–34.63‰), and the SBT range was narrower (16.79–21.36 °C) from summer to autumn (Figure 2). The surface and bottom DO values were lowest in summer, with higher values at the sea surface (4.77–6.43 mg/L) than at the bottom (2.51–6.65 mg/L) (Table 3). The sea surface DO concentrations were similar in spring (7.94–8.56 mg/L) and winter (7.40–8.00 mg/L) (Table 3).

## 4. Discussion

The life history characteristics of *S. esculenta* indicate a lifespan of approximately one year, as determined by Sun (1993) [36], who summarized the relationship between carcass length and day age. It has been reported that parent groups form groups for spawning in coastal areas from May to July, with the larvae born in June to August, and the juveniles grow and develop in the offshore area from July to October [37]. Through observations made in indoor rearing experiments, Wei et al. (1964) also confirmed that the parents died in July [38]. The duration of grouping and the time that grouping began for this species were different in the areas surveyed in the present study. For example, the grouping period in the south coastal areas of Zhejiang is April to May [39], but it is May to June in Zhoushan and the Shengsi Islands in northern Zhejiang [40]. The grouping time is mid-April to the end of May in the offshore area of Rizhao; early May to mid-July in the coastal areas of Qingdao [19,23]; early March to mid-June in Tokyo Bay [41]; the end of March to early July in Mikawa Bay, Japan [42]; mid-April to the end of May and the end of May to early June, respectively, along the south and west coasts of Korea, respectively, [43]; early May to early June in the East China Sea; and June to July in the Yellow Sea, China [44]. Generally, the grouping time is gradually delayed from south to north in the seas around China [45]. Our surveys were conducted from April to May, which was the grouping time for parent groups; August to September, which had a large abundance of newly released juveniles; November, when larger juveniles were present; and January, during the growth and development of subadults (e.g., we found the weights to be 200–400 g/ind in most stations at this time) based on an analysis of individual weights (unit: g/ind) in the East China Sea region (Figure 1h).

The groups contained juveniles that exhibited different growth rates, resulting in different resource densities. For example, the individuals at 29.00° N (164.53–210.17 g/ind at 29.00° N, 125.00–126.50° E) were larger than the individuals at 29.50° N (68.14–118.10 g/ind at 29.50° N, 124.50–126.00° E) from August to September (Figure 1f). Based on the mixed distributions of individuals with weights ranging from 100–200 to 300–400 g/ind in summer, we concluded that there was a mixed distribution of juveniles and subadult groups. Wang et al. (2019) concluded that the differences in the duration of the larvae hatching stage can lead to differences in the growth of the species [18]. Li et al. (1963) suggested that differences in the time of arrival for grouping in the coastal areas in each year might also cause differences in growth [19]. Li (1963) confirmed that a mixed distribution of subadults and adults was found in the overwintering ground of the Yellow Sea [19]. Our study found more juveniles at latitudes of 27.50–28.00° N and 29.00° N and more subadult individuals at 28.50° N in autumn.

The seasonal distribution characteristics could be explained by groups of *S. esculenta* making a planned migration from the coastal areas to the offshore areas. Li et al. (2010) observed clear seasonal variations in biomass and abundance in Jiaozhou Bay, with higher resource densities in summer and autumn than in the other two seasons [46]. Niu et al. (2017) found that the areas with a higher resource density gradually moved from Linshan Bay to the offshore area southwest of the Linshan Islands due to the growth of juveniles, with a tendency for the area with a high CPUE_w_ to move to the south and east of Linshan Bay [47]. Our study found more juveniles in coastal shallow areas and larger individuals in offshore sea areas in spring. We found more larger individuals in the northern areas than in the southern areas of the East China Sea region. In spring, the individual weight of the species decreased from 253.00 to 89.20 g/ind at 28.50°→26.50° N, 123.00° E, and the individual weight decreased from 541.00 to 109.85 g/ind at 34.00°→27.00° N, 122.00° E (Figure 1e). We, therefore, concluded that the southern and northern areas of the East China Sea region were spawning and nursery grounds, respectively, in spring.

In terms of the seasonal variations in CPUE_w_, although the average CPUE_w_ values were similar in spring and autumn, there were differences in the composition of the catches. In spring, the majority of the catches were composed of parent groups, while in autumn, the majority of the catches were composed of juvenile groups. For the areas of 32.50–35.00° N and 121.50–123.00° E in autumn, the CPUE_w_ value decreased from the north to south: 1338.00–1631.17 g/h at 34.00–35.00° N to 124.36–428.145 g/h at 32.50–33.50° N (Figure 1c). Yan et al. (2007) concluded that the location of the central distribution of this species in the East China Sea region (in the period of 2000–2001) was the area of 124.00° E to the east and both sides of 29.00° N, 124.00–125.00° E in spring, autumn, and winter [10]. However, our study suggested that the location of the central distribution was the latitude line of 28.00° N in autumn and winter. For example, larger individuals (300–400 g/ind) were found on both sides of 28.00° N in autumn (Figure 1c). The CPUE_w_ in autumn was highest at 28.00° N and decreased on both sides of the 28.00° N line, including the areas of 30.50°→28.00° N and 28.00°→27.00° N (Figure 1c). We found individuals with a weight of < 100 g/ind at 31.00° N, 125.00° E in autumn (Figure 1c), indicating the possibility of juveniles migrating from the open sea to this location, as well as the presence of larvae in the open sea in the East China Sea region. We found 100–200 g/ind at 27.00–31.00° N, 123.00–127.00° E, 200–300 g/ind at 26.50–32.00° N, 121.50–126.50° E, and 300–400 g/ind at 27.50–29.00° N, 123.50–126.00° E in autumn (Figure 1c), indicating a mixed distribution of juveniles and subadults. The individual weight increased from 2.70 to 44.97 g/ind at 28.00° N 125.00° E→30.50° N 125.50° E in winter (Figure 1h). We found larger individuals in the area south of 30.00° N in summer: 151.29 g/ind in 27.50° N 124.50–125.00° E, 174.50 g/ind in 27.00° N 123.00–124.42° E, and 284.90 g/ind in 26.50° N 123.00–123.50° E (Figure 1f). We concluded that the subadult groups might have dispersed widely for feeding and growth along the latitude of 30.00° N and to the south in summer.

We concluded that the southern area of the Yangtze River extending north was the spawning ground in spring, including the area at 29.00–34.50° N, 124.00–124.50° E, because of the similar distributions of larvae and parent groups and the occurrence of a large number of larvae at 28.00–30.00° N, 123.00–126.00° E (Figure 1e). Additionally, we concluded that the area of 28.00–30.50° N, 125.50–126.50° E was the nursery ground due to the presence of larvae with a weight of 5.23 g/ind at 30.00° N, 126.00° E in summer (Figure 1f). The CPUE_w_ values were 2873.01 and 6678.00 g/h at 30.50° N, 125.50° E and 30.50° N, 126.50° E in summer, which corresponded to individual weights of 47.03 and 39.05 g/ind, respectively (Figure 1b,f). The CPUE_w_ values of the subadult groups were 1000–1500 g/h at 34.50–35.00° N, 122.00–122.50° E, and the individual weights were 433.60, 381.20, and 292.00 g/ind at 35.00° N, 122.00° E; 34.50° N, 122.50° E; and 34.50° N, 122.00° E in autumn, respectively (Figure 1b,f). The individual weight was 22.40 g/ind at the neighboring station at 34.00° N, 122.00° E in autumn (Figure 1f). Juveniles with weights of 2.70 and 44.97 g/ind were found at 28.00° N, 125.00° E and 30.50° N, 125.50° E, respectively, indicating that these two stations were influenced by the warmer bottom water in winter (Figure 1g). According to Zhang et al. (2019), the offspring groups begin their overwinter migration to the central and southern areas of the Yellow Sea in November with the decreasing water temperature (~15.00 °C SST in the Qingdao area) [37]. They overwinter with a mixed distribution in the central and southern parts of the Yellow Sea, north of Taiwan, and southwest of Tsushima Island in winter. They then migrate radially from the overwintering grounds to the spawning grounds in the near shore area after four months of growth. Zhang et al. (2019) and the present study found that their spawning grounds included the area north of the mouth of the Yalu River and the southern part of the Zhoushan coastal waters, but they did not extend beyond the southern boundary of the Zhoushan coastal waters [37]. The spawning grounds became nursery grounds when the parent groups finished spawning. Zheng et al. (2003) concluded that their overwintering grounds included the southwestern part of Tsushima Island, central parts of the Yellow Sea (approximately 38.00–33.00° N, 122.50–123.83° E), and the area north of Taiwan [23]. Individual weights of 5.23 and 33.4 g/ind were recorded at 30.00° N, 126.00° E and 30.00° N, 124.50° E, respectively, indicating the mixed distribution of larvae and juveniles in summer (Figure 1f). Individual weights of 96.30 to 75.67 g/ind and CPUE_w_ values of 5200.20 to 1180.40 g/h were found in the area of 30.00° N 124.00°→127.00° E in summer, indicating the presence of many larger individuals in the coastal areas (Figure 1f). An individual weight of 58.38 g/ind and CPUE_w_ of 3794.70 g/h were observed at 29.00° N, 123.50° E in summer, highlighting the importance of coastal areas as nursery grounds (Figure 1b,f).

Li (1963) found that the groups of *S. esculenta* exhibited a preference for water depths of ~70.00 m during the overwintering period, but in the coastal areas, they were present at depths of ~5.00–10.00 m for long periods of time [19]. We found that the deepest distribution of *S. esculenta* in winter was 66.00 m, and there was a large depth range in spring, summer, and autumn. Zhejiang Aquatic Products Bureau (1956) showed that the most suitable water temperature for fishing was in the range of 16.00–32.00 °C in Hainan Island and Beibu Gulf of China, 10.00–24.00 °C in Kyushu of Japan and the south bank of the Korean Peninsula, and 11.00–23.00 °C in Jiaozhou Bay of China [39]. Li (1963) argued that the hatching rate of eggs could exceed 70% when the water temperature exceeded 6.00 °C, and the incubation time was 20–21 d under conditions with a salinity of 30.00 ppt and water temperature of 22.00–24.00 °C [19]. There was a large difference in the surface and bottom water temperatures in summer and a surface salinity range of 27.69–34.23‰ (Table 3), indicating substantial variability in the water conditions. In particular, some of the *S. esculenta* inhabited areas with low SSS, suggesting that their potential distribution areas might be influenced by water from the Changjiang River in summer. The range of SSTs was 16.11–20.12 °C (Table 3). The water temperature ranges in surface and bottom water were similar (SST 16.11–20.12 °C vs. SBT 15.93–19.76 °C) in winter (Table 3). Furthermore, there was a difference of 4.00 °C between the upper limit of the SBT (19.76 °C) and the lower limit of the SST (15.93 °C). The lower limit range of water temperature values (both SST and SBT) during winter was 15.93 °C to 16.11 °C, indicating relatively consistent minimum temperatures in both surface and bottom waters (Table 3). Thus, we concluded that the overwintering ground might be influenced by the Kuroshio warm current, and the groups of *S. esculenta* preferred to stay in areas with a stable water temperature of ~20.00 °C. The most suitable sea bottom salinity for fishing was similar in spring and autumn, but the difference in the CPUE_w_ between these two seasons was large. The upper limit of sea surface salinity was similar (34.23–34.52‰) throughout the year (Table 3), indicating that some groups preferred to stay in areas with a high surface salinity. We found that the most suitable salinity for this species was 31.50–34.50‰ (Figure 4). Tian et al. (2008) concluded that the fishery production of *S. esculenta* substantially decreased in the late 1980s in Korea due to the increase in winter SSTs in the Yellow Sea [48]. Uda (1931) found that the population of *S. esculenta* preferred to remain in an 8–9° narrow and long isothermal area that began from the 36.00° N latitude line and extended northwestward to the south bank of the Shandong Peninsula and extended southeastward to the south bank of the Korean Peninsula [49]. Wang et al. (2019) suggested that larvae could actively avoid the negative influences of high water temperatures in the coastal waters in later periods, and recently hatched larvae might have a longer favorable growing period [18].

The role of the seasons for *S. esculenta* was identified as overwintering in winter to spring and spawning and nurseries in summer to autumn, with the spawning and nursery grounds located in the same sea area. This suggests that *S. esculenta* utilizes different seasonal periods for specific stages of its life history. Additionally, there was a large sea bottom area with a low DO content (minimum of 2.51 mg/L) in summer (Table 3), and many *S. esculenta* juveniles might have adapted to endure the negative influence of the low oxygen content. Water depth had an important impact on this species over the one year of its life history, and large groups of *S. esculenta* were found in the coastal area in autumn and in deeper offshore areas in winter. We proposed the following fisheries management strategies. (1) Species-specific management measures should be enforced to manage the populations of Chinese coastal cephalopods in overexploited and degraded ecosystems, with a particular focus on the monitoring of recruitment groups. (2) It is important to monitor the water temperature from May to August over the long term. (3) It is important to monitor the relationship between the populations of coastal cephalopods and other fish species. Long-term fishing pressure has reduced the population of many fish species, with cephalopods, including *S. esculenta*, using vacant ecological niches to increase their population [7]. (4) It is important to delineate the management units and perform the necessary actions, including stock releases (usually in late July). The released groups will grow quickly in the coastal area and form a large recruitment population. The remaining groups will become a reproductive population after migrating over winter and will then supplement the natural population.

## 5. Conclusions

The main conclusions of this study were as follows: (1) The location of the central distribution of the groups of *S. esculenta* from 2018 to 2019 was the latitude line of 28.00° N, which was more northerly than reported by Yan et al. (2007) [10]. We propose that a gradient of rising water temperature could potentially force a northward shift in the location of its central distribution. In the future, in addition to monitoring water temperature, it will also be important to monitor environmental factors such as SSS, wind direction, seabed topography, and the presence of prey organisms. It is necessary to create a database and conduct population classifications, including gene communication and genetic exchange of different groups in geographical areas. The useful information obtained in this study will offer insights into how to fine-tune conservation and fishery management measures for this species and the wider resource in the future.

## Figures and Tables

**Table 1:** Details of the cruise number and season with survey time and total sampling stations.

Cruise Number and Seasons	Survey Time	Total Sampling Stations
First: Autumn	2–11 November 2018	127
Second: Winter	4–27 January 2019	111
Third: Spring	22 April–10 May 2019	141
Fourth: Summer	13 August–27 September 2019	140

**Table 2:** The value and value range of average catch per unit effort by weight (CPUE_w_) at all stations (unit: g/h), average CPUE_w_ at collection stations (unit: g/h), value range of CPUE_w_ (unit: g/h), average CPUE_n_ at all stations (unit: ind/h), average CPUE_n_ at collection stations (unit: ind/h), the value range of CPUE_n_ (unit: ind/h), average AIW (unit: g/ind), value range of AIW (unit: g/ind), most suitable distribution area, and the statistical details of station number classified by AIW (0–100, 100–200, 200–300, 300–400, 400–500, >500; unit: g/ind) in spring to winter.

	Spring	Summer	Autumn	Winter
Average CPUE_w_ at all stations	111.57	423.44	247.04	58.55
Average CPUE_w_ at collection stations	683.99	1297.94	922.75	504.45
Value range of CPUE_w_	46.80–2705.00	59.40–6678.00	88.24–4346.00	2.70–932.36
Average CPUE_n_ at all stations	0.48	5.68	1.51	0.19
Average CPUE_n_ at collection stations	2.93	17.40	5.63	1.64
value range of CPUE_n_	0.86–9.60	1.00–171.00	1.00–29.00	0.98–3.00
Average AIW	313.91	117.08	213.39	291.96
Value range of AIW	4.88–819.05	5.23–360.87	22.40–495.30	2.70–403.29
Most suitable distribution area	122.00–124.00° E 26.50–27.50° N	122.00–127.00° E 26.50–31.00° N	121.50–122.00° E 33.00–34.50° N	121.00–127.00° E 26.50–32.00° N
Groups classified by AIW	Station number			
0–100 g/ind	2	24	4	2
100–200 g/ind	2	15	12	1
200–300 g/ind	10	6	11	2
300–400 g/ind	4	1	5	6
400–500 g/ind	1	0	2	2
>500 g/ind	4	0	0	0

**Table 3:** The measured in situ value range of environmental factors in spring to winter: depth (m), sea surface temperature SST (°C), sea bottom temperature SBT (°C), sea surface salinity SSS (‰), sea bottom salinity (‰), sea surface dissolved oxygen SSDO (mg/L), and sea bottom dissolved oxygen SBDO (mg/L). The superscript of the values is the proportional deviation calculated by the *HSI* model, and the proportional deviation of depth, SST, SBT, SSS, and SBS sum up to 100.00%. A higher proportional deviation means a higher impact of environmental factors on the distribution of the species.

	Spring	Summer	Autumn	Winter
Depth	13.00–120.00^8.66%^	10.00–133.00^10.41%^	14.00–115.00^27.86%^	66.00–107.00^26.16%^
SST	13.17–25.78^12.01%^	26.20–29.61^4.60%^	16.91–25.16^17.67%^	16.11–20.12^29.94%^
SBT	9.60–20.53^13.77%^	13.09–28.25^30.35%^	10.08–22.83^14.99%^	15.93–19.76^19.53%^
SSS	30.66–34.52^46.17%^	27.69–34.23^25.14%^	30.95–34.45^21.44%^	33.68–34.39^11.50%^
SBS	31.53–34.80^19.39%^	32.95–34.68^29.50%^	31.51–34.77^18.04%^	33.82–34.51^12.87%^
SSDO	7.94–8.56	4.77–6.43		7.40–8.00
SBDO	7.94–9.24	2.51–6.65		7.45–8.02

## Data Availability

Data are contained within the article.

## References

[1] Hunsicker M.E., Essinaton T.E., Watson R., Sumaila U.R. (2010). The contribution of cephalopods to global marine fisheries: Can we have our squid and eat them too?. Fish Fish..

[2] Xavier J.C., Phillips R.A., Cherel Y. (2011). Cephalopods in marine predator diet assessments: Why identifying upper and lower beaks is important. ICES J. Mar. Sci..

[3] Ye Y., Cochrane K. (2011). Global overview of marine fishery resources. Review of the state of world marine fishery resources. FAO Fish. Aquac. Tech. Pap..

[4] Quetglas A., Keller S., Massutí E. (2015). Can Mediterranean cephalopod stocks be managed at MSY by 2020? The Balearic Islands as a case study. Fish. Manag. Ecol..

[5] Arkhipkin A.I., Rodhouse P.G., Pierce G.J., Sauer W., Sakai M., Allcock L. (2015). World Squid Fisheries. Rev. Fish. Sci. Aquac..

[6] FAO (2022). The State of World Fisheries and Aquaculture 2022. Towards Blue Transformation.

[7] Caddy J.F., Rodhouse P.G. (1998). Cephalopod and groundfish landings: Evidence for ecological change in global fisheries. Rev. Fish Biol. Fishe..

[8] Pecl G.T., Jackson G.D. (2008). The potential impacts of climate change on inshore squid: Biology, ecology and fisheries. Rev. Fish Biol. Fish..

[9] Wu Q., Wang J., Li Z.Y., Dai F.Q., Chen R.S., Sun S., Jin X.S. (2015). The community structure and biodiversity of cephalopoda in central and southern Yellow Sea. Mar. Sci..

[10] Yan L.P., Li S.F., Ling J.Z., Zheng Y.J. (2007). Study on the resource alteration of commercial cuttlefish in the East China Sea. Mar. Sci..

[11] Okutani T. (1995). Cuttlefish and Squids of the World in Color.

[12] Dong Z.Z. (1991). Biology of Economic Cephalopods in World Oceans.

[13] Cheng J.S., Zhu J.S. (1997). Feeding characteristics and trophic levels of main economic invertebrates in the Yellow Sea. Acta Oceanol. Sin..

[14] Zheng X., Ikeda M., Kong L. (2010). Genetic diversity and population structure of the golden cuttlefish, *Sepia esculenta* (Cephalopoda: Sepiidae) indicated by microsatellite DNA variations. Mar. Ecol..

[15] Watanuki N., Iwashita T., Kawamura G. (2000). Cuttlefish spawning and visually mediated entry into basket traps. Fish. Sci..

[16] Jereb P., Roper C.F.E. (2005). Cephalopods of the World. An annotated and illustrated catalogue of cephalopod species known to date. Volume 1. Chambered Nautiluses and Sepioids.

[17] Arima S., Hiramatsu T., Tako N. (1962). Mass production of deedling and maintenance of Sepiidae–I. Annu. Res. Rep. Buzen Fish..

[18] Wang L.L., Zhang X.M., Wang Z., Song N., Gao T.X. (2019). Morphological characteristics and genetic differentiation of a breeding population of *Sepia esculenta* in Qingdao. J. Fish. Sci. China.

[19] Li J.Y. (1963). On the breeding and migration of the golden cuttlefish, *Sepia esculenta* Hoyle, living in Yellow Sea. Period. Ocean Univ. China.

[20] Zhang X., Qi Z.Y., Li J.M. (1962). China Economic Zoology–Marine Mollusc.

[21] Zhao R.Y., Cheng J.M., Zhao D.D. (1982). Dalian Marine Mollusca Flora.

[22] Qi Z.Y., Ma J.T., Wang Z.R., Lin G.Y., Xu F.S., Dong Z.Z., Li F.L., Lv D.H. (1989). Mollusca of Huanghai and Bohai.

[23] Zheng Y.J., Chen X.Z., Cheng J.H. (2003). Biological Resources and Environment of East China Sea Continental Shelf.

[24] Natsukari Y., Tashiro M. (1991). Neritic squid resources and cuttlefish resources in Japan. Mar. Behav. Physiol..

[25] Robinson C.J., Gómez-Gutiérrez J., de León D.A.S. (2013). Jumbo squid (*Dosidicus gigas*) landings in the Gulf of California related to remotely sensed SST and concentrations of chlorophyll a (1998–2012). Fish. Res..

[26] Pang Y., Tian Y., Fu C., Wang B., Li J., Ren Y., Wan R. (2018). Variability of coastal cephalopods in overexploited China Seas under climate change with implications on fisheries management. Fish. Res..

[27] Belkin I.M. (2009). Rapid warming of large marine ecosystems. Prog. Oceanogr..

[28] Cai W., Borlace S., Lengaigne M., Van Rensch P., Collins M., Vecchi G., Timmermann A., Santoso A., McPhaden M.J., Wu L. (2014). Increasing frequency of extreme El Niño events due to greenhouse warming. Nat. Clim. Change.

[29] Cai W., Wang G., Santoso A., McPhaden M.J., Wu L., Jin F.F., Timmermann A., Collins M., Vecchi G., Lengaigne M. (2015). Increased frequency of extreme La Niña events under greenhouse warming. Nat. Clim. Change.

[30] Wang K.Y., Lee K.T., Liao C.H. (2010). Age, growth and maturation of swordtip squid (*Photololigo edulis*) in the southern East China Sea. J. Mar. Sci. Technol..

[31] Wang K.Y., Chang K.Y., Liao C.H., Lee M.A., Lee K.T. (2013). Growth strategies of the swordtip squid, *Uroteuthis edulis*, in response to environmental changes in the southern East China sea–a cohort analysis. Bull. Mar. Sci..

[32] Yamaguchi T., Kawakami Y., Matsuyama M. (2015). Migratory routes of the swordtip squid *Uroteuthis edulis* inferred from statolith analysis. Aquat. Biol..

[33] Crozier L.G., Hutchings J.A. (2014). Plastic and evolutionary responses to climate change in fish. Evol. Appl..

[34] Chang Y.J., Sun C.L., Chen Y., Yeh S.Z., Dinardo G. (2012). Habitat suitability analysis and identification of potential fishing grounds for swordfish, *Xiphias gladius*, in the South Atlantic Ocean. Int. J. Remote Sens..

[35] Vincenzi S., Caramori G., Rossi R., De Leo G.A. (2006). A GIS–based habitat suitability model for commercial yield estimation of *Tapes philippinarum* in a Mediterranean coastal lagoon (Sacca di Goro, Italy). Ecol. Model..

[36] Sun G., Lan Y.R. (1993). On fishery, ecology and resource of Sepiidae in Japan. Mod. Fish. Inform..

[37] Zhang X.M., Wang L.L., Zhang Y.Y., Luo G., Gao H.Y. (2019). Strategy optimization of stock enhancement of golden cuttlefish, (*Sepia esculenta*) based on structural characteristics of reproductive and recruitment populations. J. Fish. China.

[38] Wei Z.B. (1964). Preliminary observation of behaviors and biological traits of golden cuttlefish. Chin. J. Zool..

[39] Zhejiang Aquatic Bureau (1992). The Materials of Aquatic Works: Introductions of Dachenyang Moyu Fishing Grounds.

[40] Huang Z.H. (1952). Cuttlefish industry in Shengsi islands. Huadong Fish..

[41] Koido Y., Kurata Y., Kawakami T. (1956). Ecology on *Sepia esculenta* and *Sepiella japonica* caught in Tokyo Bay. Aquaculture.

[42] Yasuda J. (1951). Some ecological notes on the cuttlefish, *Sepia esculenta* Hoyle. Nippon Suisan Gakkaishi.

[43] Yamamoto T. (1942). Embryonal development of *Sepia esculenta* Hoyle. Bot. Zool..

[44] Okamura O., Yamada U. (1986). Fishes of the East China sea and the Yellow Sea. Contrib. Seikai Reg. Fish. Res. Lab..

[45] Hao Z.L., Zhang X.M., Zhang P.D. (2007). Biological characteristics and multiplication techniques of *Sepia esculenta*. Chin. J. Ecol..

[46] Li C.L., Zhao B., Hu W., Li Q.C., Song A.H., Zhao Y.F. (2010). Seasonal biomass variations of *Sepia esculenta* in Jiaozhou Bay of Yellow Sea. J. Yantai Univ. (Nat. Sci. Eng. Ed.).

[47] Niu C., Zhang X.M., Ding P.W., Shan B.B. (2017). Preliminary assessments on growth characteristics, resource distribution and *Sepia esculenta* releasing effect in Jiaonan coastal water. Period. Ocean Univ. China.

[48] Tian Y., Kidokoro H., Watanabe T., Iguchi N. (2008). The late 1980s regime shift in the ecosystem of Tsushima warm current in the Japan/East Sea: Evidence from historical data and possible mechanisms. Prog. Oceanogr..

[49] Uda M. (1931). Of the monthly oceanographical charts of the adjacent seas of Japan based on the averages for the 13 years from 1918–1930, with a discussion of the current system inferred from these charts. Part 2: From January to June.. J. Imp. Fish. Exp. St..

